# Bevacizumab and gefitinib enhanced whole-brain radiation therapy for brain metastases due to non-small-cell lung cancer

**DOI:** 10.1590/1414-431X20176073

**Published:** 2017-11-17

**Authors:** R.F. Yang, B. Yu, R.Q. Zhang, X.H. Wang, C. Li, P. Wang, Y. Zhang, B. Han, X.X. Gao, L. Zhang, Z.M. Jiang

**Affiliations:** 1 Department of Thoracic Surgery, Qianfoshan Hospital of Shandong Province, Shandong University, Ji'nan, Shandong, China; 2 Department of Thoracic Surgery, Taian City Central Hospital, Taian, Shandong, China; 3 Department of Anus and Intestine Surgery, Taian City Central Hospital, Taian, Shandong, China; 4 Department of Digestive System, Taian City Central Hospital, Taian, Shandong, China

**Keywords:** Non-small-cell lung cancer, Brain metastasis, Bevacizumab, Gefitinib, Whole brain radiotherapy

## Abstract

Non-small-cell lung cancer (NSCLC) patients who experience brain metastases are usually associated with poor prognostic outcomes. This retrospective study proposed to assess whether bevacizumab or gefitinib can be used to improve the effectiveness of whole brain radiotherapy (WBRT) in managing patients with brain metastases. A total of 218 NSCLC patients with multiple brain metastases were retrospectively included in this study and were randomly allocated to bevacizumab-gefitinib-WBRT group (n=76), gefitinib-WBRT group (n=77) and WBRT group (n=75). Then, tumor responses were evaluated every 2 months based on Response Evaluation Criteria in Solid Tumors version 1.0. Karnofsky performance status and neurologic examination were documented every 6 months after the treatment. Compared to the standard WBRT, bevacizumab and gefitinib could significantly enhance response rate (RR) and disease control rate (DCR) of WBRT (P<0.001). At the same time, RR and DCR of patients who received bevacizumab-gefitinib-WBRT were higher than those who received gefitinib-WBRT. The overall survival (OS) rates and progression-free survival (PFS) rates also differed significantly among the bevacizumab-gefitinib-WBRT (48.6 and 29.8%), gefitinib-WBRT (36.7 and 29.6%) and WBRT (9.8 and 14.6%) groups (P<0.05). Although bevacizumab-gefitinib-WBRT was slightly more toxic than gefitinib-WBRT, the toxicity was tolerable. As suggested by prolonged PFS and OS status, bevacizumab substantially improved the overall efficacy of WBRT in the management of patients with NSCLC.

## Introduction

Lung cancer is a major cause for cancer-related deaths and up to 222,520 new cases were diagnosed in the US in 2010 (1). Almost 85% of lung cancer cases are classified as non-small cell lung cancer (NSCLC), which includes both non-squamous carcinoma and squamous cell carcinoma (2). Although substantial improvement in treatments has been made, NSCLC prognosis remains unfavorable since it is an uncontrolled systemic disease. Moreover, about 20 to 40% NSCLC patients may end up with brain metastases (BM) as disease progresses (3). Cross-sectional studies have identified several risk factors for NSCLC metastases among which lymphovascular space invasion, younger age and larger tumor size have been considered significant (4). To date, conventional treatments for brain metastases resulting from NSCLC include whole-brain radiation therapy (WBRT), surgical resection, stereotactic radiosurgery or the combination of these approaches (5). A randomized phase III trial on NSCLC patients who had BM suggested that WBRT could increase median survival time to roughly 4.2 months (6) and they also indicated that the effectiveness of WBRT was strongly associated with patient age and number/location of metastatic lesions (7–10).

The epidermal growth factor receptor (EGFR) is highly stimulated in epithelial cancers, such as NSCLC (11). Increasing evidence indicates that NSCLC patients exhibit higher EGFR levels and they may have a lower risk of BM if EGFR inhibitors (e.g., gefitinib) are introduced in the treatment (12). It is estimated that about 10∼15% NSCLC patients possess EGFR mutations (e.g., exon 19 deletions or the L858R point mutation) and they could largely benefit from gefitinib (13). Currently, the feasibility of introducing EGFR inhibitors into WBRT is being studied in many clinical trials, and gefitinib is one that is able to penetrate the blood-brain barrier through the concurrent WBRT therapy (14,15).

Besides, vascular endothelial growth factor (VEGF) is another over-expressed biomarker in BM of NSCLC (16). Therefore, VEGF could serve as a crucial therapeutic target for BM of NSCLC. Accordingly, attention has been turned to the anti-VEGF monoclonal anti-bodies, such as bevacizumab. In fact, bevacizumab was not accepted for intracranial lesions of NSCLC patients due to hemorrhage at first (17). However, later multiple documentations from a large sample (i.e., 10,000) of patients with primary cancers and brain metastases indicated that the risks of cerebral hemorrhage were similar between treatments with and without bevacizumab (18). Notably, bevacizumab mainly functions to block the combination of VEGF and VEGFR, thereby lessening formation of new blood vessels (19). Furthermore, bevacizumab also seems to normalize aberrant blood vessels, and it boosts the concentration of antineoplastic agents within tumor tissues through enhancing permeability of the vessels. Based on the above strengths of bevacizumab, it has been investigated broadly and deeply in treating active brain metastatic lesions from NSCLC (20). Owing to the particularity of BM, the combined therapy of bevacizumab and WBRT was also assessed regarding their treatment efficacy in managing BM from solid tumors (21).

Nevertheless, up to now, only a few studies combined bevacizumab, gefitinib and WBRT for treating BM from NSCLC. Therefore, this study was designed to explore the synthetic efficacy of bevacizumab, gefitinib and WBRT for NSCLC patients with BM.

## Patients and Methods

### Patients

This retrospective study included 228 patients who were recruited from Qianfoshan Hospital of Shandong Province between March 2008 and March 2014. The patients were randomly allocated to 3 groups, and they received treatments of bevacizumab+gefitinib+WBRT (n=76), gefitinib +WBRT (n=77), and WBRT (n=65). The patients were included if: 1) their primary lesions were histologically confirmed as NSCLC; 2) their BM was diagnosed by magnetic resonance imaging, and at least one measurable intracranial metastases could be utilized to assess treatment efficacy; 3) they were treated with WBRT due to inability and rejection to receive surgical treatments, and their lesions displayed no acute bleeding; 4) their hemogram had no obvious abnormity; 5) their Karnofsky performance status (KPS) scoring achieved h 60 scores; and 6) their medical records were complete. The subjects were excluded when: 1) they had other tumor lesions, apart from primary lesions and brain metastasis; 2) unbearable toxic side effects were present during treatments; and 3) patients had any other malignant disease or neurologic disease, such as the Alzheimer's disease. Detailed clinical information of the three treatment groups are disclosed in Table 1.

**Table 1.** Clinical characteristic of non-small-cell lung cancer patients with brain metastases treated with bevacizumab+gefitinib+WBRT, gefitinib+WBRT and WBRT.

Characteristics	Bevacizumab+gefitinib+WBRT	Gefitinib+WBRT	WBRT	P
Age (years old)	58.42±14.88	60.64±13.57	58.78±10.92	0.470*
Gender				
Male	35	29	35	0.129#
Female	41	48	40	
KPS	59.41±5.50	61.00±5.31	60.07±5.95	0.249*
Smoking				
Never or light	42	43	39	0.830#
Heavy	34	34	26	
Tumor histology				
Adenocarcinoma	48	52	38	0.153#
Squamous	8	14	15	
Large cell	20	11	12	
Number of brain metastases				
≤5	40	38	34	0.906#
>5	36	39	31	
ECOG/PS				
0	15	13	16	0.780#
1/2	61	64	59	
EGFR mutation status				
Del	43	40	38	0.686#
L858R	29	30	28	
Others	4	7	9	

WBRT: Whole brain radiotherapy; KPS: Karnofsky performance status; ECOG/PS: Eastern Cooperative Oncology Group Performance Scale.

* Kruskal-Wallis test;

# Chi-square test.

### Study design and treatment

This study was approved by the Qianfoshan Hospital of Shandong Province Ethics Committee and was conducted based on approved guidelines provided by Qianfoshan Hospital of Shandong Province (approval No. CNSDQFSH010). All participants signed and submitted the written informed consent before treatment commencement.

#### WBRT group

Patients were treated by standard WBRT delivered with 3-Gy per day by fractions, 5 days per week until the total dosage of 30 Gy was achieved. Treatment was delivered using linear accelerator in which energy was set between 4 and 8 MV photons. After patients were treated, potential neurotoxicity was identified in 3 patients and the dosage of WBRT was changed to 2.5 Gy with a total of 14 fractions.

#### Gefitinib-WBRT group

Gefitinib at a dosage of 250 mg/day was administered to 76 patients over a period of 6 days after enrollment. Then, WBRT was carried out in conjunction with gefitinib at a dosage of 250 mg/day. The dose of concomitant WBRT was 40 Gy in 20 fractions. These procedures were interrupted when severe adverse effects or disease progression occurred. Dosage was reduced by 50 or 100 mg if there was a presence of severe adverse effects (grade 3), such as diarrhea and rash.

#### Bevacizumab-gefitinib-WBRT group

Bevacizumab (5 mg/kg) was diluted with 0.9% normal saline (volume: 100 mL), and an intravenous drip was performed once every 14 days. If the first intravenous drip that lasted for 90 min was characterized by fine tolerance, the second one would done with a duration of <60 min and the following ones with a duration <30 min. Other treatments were consistent with gefitinib-WBRT group.

All patients were accompanied by bevacizumab chemotherapy, administered orally with the dosage of 200 mg·(m^2^)^-1^·day^-1^ over a period of 5 days. Anti-emetics were systematically used before bevacizumab was administered. Treatments were cycled every 28 days. Patients were continuously administered with concomitant treatments including anti-epileptic drugs, anti-emetics, mannitol and corticosteroids. All medications were strictly guided by physicians.

### Examination of EGFR mutations

About 25∼30 mg tumor tissues were extracted and mechanically sheared. The E.Z.N.A™ Tissue DNA Kit (Omega Corporation, USA) was applied to extract DNA, and the experimental procedures were all in accordance with the operating instructions. Polymerase chain reaction (PCR) was adopted to amplify 4 exons of EGFR (i.e., 18, 19, 20, and 21), with primer sequences shown in Supplementary Table S1. The PCR reaction system (total volume: 20 µL) consisted of HotStar-Taq buffer, 2.0 mmol/L Mg^2+^, 0.2 mmol/L dNTP, 0.2 μmol/L upstream primer, 0.2 μmol/L downstream primer, 1 u HotStar-Taq polymerase (Qiagen Inc., Germany) and 10 ng DNA template. The PCR reaction condition was summarized as: 35 cycles of 94°C (15 s), 56°C (30 s), and 72°C (1 min), and final 72°C (2 min) for extension. After PCR products were purified, the DNA sequencer (ABI 3130xl, Applied Biosystems, USA) was employed for sequential analysis. The results were analyzed by Polyphred software (University of Washington, USA), and differences were drawn after comparing the sequencing results and EGFR gene sequences in the genebank (NM_005225.3).

### Patient evaluation

Tumor response was evaluated every 2 months based on the Response Evaluation Criteria in Solid Tumors (RECIST) version 1.0 (22). Complete response (CR) was confirmed when brain metastasis totally disappeared and no new lesions appeared for at least 4 weeks. Partial response (PR) was determined when the product of tumor horizontal diameter and vertical diameter diminished more than 50%. Stable disease (SD) was ascertained if the product of tumor diameters lessened less than 50%, and enlarged less than 25%. Progressive disease (PD) was determined if the product of tumor diameters increased more than 25% or new lesions appeared. Then, the evaluation criterion of response rate (RR) was formed by combining CR and PR, whereas disease control rate (DCR) was calculated by combining CR, PR and SD. Adverse events were evaluated once a month during the follow-up period based on the National Cancer Institute Common Toxicity Criteria (NCICTC) version 2.0. Cognitive testing (23) was also performed for patients on the 14th day after WBRT as well as on each follow-up visiting day. Progression free survival (PFS) was defined as time between treatment and when clinical progression was registered or when the patient died. Overall survival (OS) time was defined between treatment time and time when patients died.

### Statistical analysis

We used one-way ANOVA (Kruskal-Wallis test) and chi-square test to analyze differences in demographic characteristics and clinical data among the three groups. Logistic regression was used to determine independent factors that affect RR or DCR. The Kaplan-Meier method was used to plot survival curves, which compare both OS and PFS among the three groups. Statistical significance was defined by two-sided P-value of less than 0.05 and all statistical analysis was implemented using SPSS 21 software (USA).

## Results

### Demographic features of patients

Table 1 shows the detailed baseline characteristics of the three groups. Distribution of clinical characteristics was balanced among the bevacizumab-gefitinib-WBRT, gefitinib-WBRT and WBRT groups. The mean age, the proportion of male and female patients and KPS scores in the above three groups were similar. Besides, smoking status, tumor histology, number of brain metastasis, EGFR mutation statuses were well matched among the three groups (all P>0.05).

### Short-term treatment effects

The treatment response of three groups is shown in Table 2. PR, PD, RR and DCR had significant differences among three groups. The RR and DCR in the bevacizumab-gefitinib-WBRT group were 80.3 and 96.1%, respectively. The overall RR and DCR in the gefitinib-WBRT group were 70.1 and 84.3%, respectively. The WBRT group exhibited an RR and DCR of 44.0 and 60.0%, respectively. Furthermore, the bevacizumab-gefitinib-WBRT group obtained higher DCR than gefitinib-WBRT.

**Table 2.** Responses in patients with brain metastases due to non-small-cell lung cancer treated with bevacizumab+gefitinib+WBRT, gefitinib+WBRT and WBRT.

Response	Bevacizumab+gefitinib+WBRT	Gefitinib+WBRT	WBRT	χ^2^	P
Response (n, %)					
Complete response	7 (9.2%)	6 (7.8%)	4 (5.3%)	0.84	0.657
Partial response	54 (71.1%)*	48 (62.3%)#	29 (38.7%)	17.33	<0.05
Stable disease	12 (15.8%)	10 (12.9%)	12 (16.0%)	0.34	0.843
Progressive disease	3 (3.9%)*	9 (11.7%)#	20 (26.7%)	16.68	<0.05
Response rate (n, %)	61 (80.3%)*	54 (70.1%)#	33 (44.0%)	23.18	<0.05
Disease control rate (n, %)	73 (96.1%)* +	64 (83.1%)#	45 (60.0%)	31.25	<0.05

* P<0.05, comparison between bevacizumab+gefitinib+WBRT and WBRT groups;

# P<0.05, comparison between gefitinib+WBRT and WBRT groups;

+ P<0.05, comparison between bevacizumab+gefitinib+WBRT and gefitinib+WBRT groups. WBRT: whole brain radiotherapy.

### Long-term treatment effects

**Figure 1.**
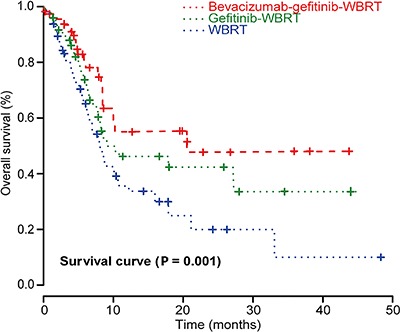
Kaplan-Meier estimation of overall survival rate in patients with brain metastases due to non-small-cell lung cancer treated with bevacizumab-gefitinib-WBRT, gefitinib-WBRT and WBRT. WBRT: whole brain radiotherapy.

**Figure 2.**
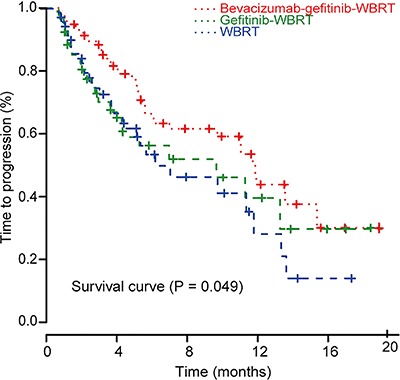
Kaplan-Meier estimation of progression-free survival in patients with brain metastases due to non-small-cell lung cancer treated with bevacizumab-gefitinib-WBRT, gefitinib-WBRT and WBRT. WBRT: whole brain radiotherapy.

**Figure 3.**
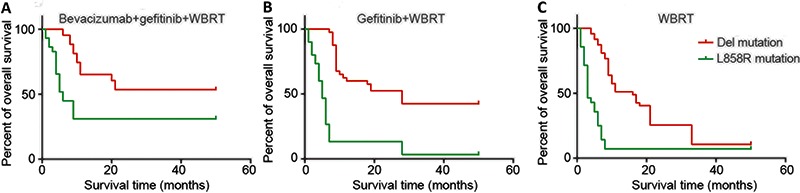
Kaplan-Meier estimation of overall survival rate in patients with brain metastases due to non-small-cell lung cancer with and without *EFGR* mutations treated with bevacizumab-gefitinib-WBRT, gefitinib-WBRT and WBRT. WBRT: whole brain radiotherapy.

**Figure 4.**
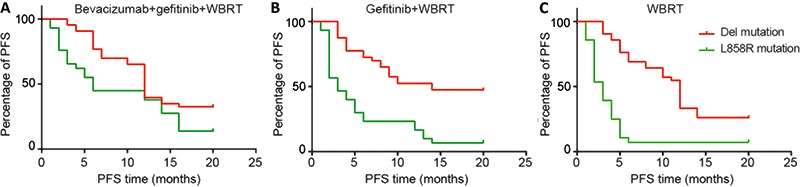
Kaplan-Meier estimation of progression-free survival in patients with brain metastases due to non-small-cell lung cancer with and without *EFGR* mutations treated with bevacizumab-gefitinib-WBRT, gefitinib-WBRT and WBRT. WBRT: whole brain radiotherapy.

Results from survival analyses for the three groups are reported in Figures 1 and 2. The median OS and PFS time were both significantly different among the three groups (P<0.05), and patients in the bevacizumab-gefitinib-WBRT group had the most favorable survival status with respect to both PFS and OS (P<0.05). The OS rates in the bevacizumab-gefitinib-WBRT, gefitinib-WBRT and WBRT groups were 48.6, 36.7, and 9.8%, respectively. The median PFS rates in the bevacizumab-gefitinib-WBRT, gefitinib-WBRT and WBRT groups were 29.8, 29.6, and 14.6%, respectively (Figure 2). Irrespective of treatment group, subjects carrying Del mutations generally possessed longer OS and PFS than those carrying L858R mutations (Figures 3 and 4).

### Side effects

The various side effects from treatments are reported in Table 3 (P>0.05). For instance, rash was the most common side effect, which accounted for 57 (75.0%), 50 (64.9%) and 45 cases (60.0%) in the bevacizumab-gefitinib-WBRT, gefitinib-WBRT and WBRT groups, respectively (P=0.137). The prevalence of nausea and vomiting in the bevacizumab-gefitinib-WBRT, gefitinib-WBRT and WBRT groups were 23.6, 18.2, and 16.0%, respectively, without a significant difference (P=0.467).

**Table 3.** Rate of adverse events in patients with brain metastases due to non-small-cell lung cancer treated with bevacizumab+gefitinib+WBRT, gefitinib+WBRT and WBRT.

Adverse events	Bevacizumab+gefitinib+WBRT	Gefitinib+WBRT	WBRT	χ^2^	P
Rash	57 (75.0%)	50 (64.9%)	45 (60.0%)	3.98	0.137
Hypertension	43 (56.6%)	41 (53.2%)	40 (53.3%)	0.22	0.895
Proteinuria	39 (43.4%)	40 (51.9%)	35 (46.7%)	0.50	0.778
Diarrhea	23 (30.3%)	25 (32.5%)	18 (24.0%)	1.42	0.492
Nausea/vomiting	18 (23.6%)	14 (18.2%)	12 (16.0%)	1.52	0.467
Headache	15 (19.7%)	13 (16.9%)	10 (13.3%)	1.12	0.572
Pneumonitis	1 (1.3%)	1 (1.2%)	0 (0.0%)	0.99	0.61
Colonic perforation	1 (1.3%)	1 (1.2%)	0 (0.0%)	0.99	0.61
Intracranial hemorrhage	1 (1.3%)	0 (0.0%)	0 (0.0%)	2.01	0.366

WBRT: Whole brain radiotherapy.

Regarding determination of the myelo-suppressive conditions (Table 4), abnormal leukocytes of the bevacizumab-gefitinib-WBRT group appeared more severe than of the gefitinib-WBRT group, which was much more serious than the WBRT group (P<0.05). The prevalence of abnormal hemoglobin seemed to be higher than in the gefitinib + WBRT group (P<0.05). Since side effects were present in the above two treatment groups, dosages of bevacizumab and gefitinib were reduced for safety purposes.

**Table 4.** Myelo-suppressive conditions in patients with brain metastases due to non-small-cell lung cancer treated with bevacizumab+gefitinib+WBRT, gefitinib+WBRT and WBRT.

Myelo-suppressive conditions	Grade 0	Grade I	Grade II	Grade III	Grade IV	χ^2^	P
Leukocyte							
Bevacizumab+gefitinib+WBRT	10	22	33	11	0	27.70	<0.05+
Gefitinib+WBRT	31	30	8	8	0	12.17	0.007#
WBRT	45	26	4	0	0	56.33	<0.05*
Hemoglobin							
Bevacizumab+gefitinib+WBRT	52	14	7	3	0	1.48	0.687+
Gefitinib+WBRT	55	12	9	1	0	1.79	0.617#
WBRT	59	9	7	0	0	4.52	0.210
Platelet							
Bevacizumab+gefitinib+WBRT	62	8	6	0	0	0.57	0.751
Gefitinib+WBRT	63	6	8	0	0	3.89	0.143
WBRT	65	8	2	0	0	2.06	0.356

Data are reported as absolute numbers. WBRT: whole-brain radiotherapy.

* P<0.05, comparison between bevacizumab+ gefitinib+WBRT and WBRT groups;

# P<0.05, comparison between gefitinib+WBRT and WBRT groups;

+ P<0.05, comparison between bevacizumab+gefitinib+WBRT and WBRT groups. WBRT: Whole brain radiotherapy.

## Discussion

Although surgical excision has been considered an effective approach for managing BM resulting from NSCLC, the median survival time for patients who experience such a disease progression is less than 3 months (24). As a standard treatment for BM caused by NSCLC, WBRT has improved the median survival time to approximately 5 months (25). Gefitinib demonstrated to be effective since it prolonged the median survival time to 9∼13.5 months for those patients (26–29). However, it is still challenging to evaluate whether introducing bevacizumab and gefitinib improves the survival status due to the lack of evidence.

This study assessed the effectiveness and tolerance of three treatments for managing patients with BM resulting from NSCLC. Our results indicated that the median OS in both the bevacizumab-gefitinib-WBRT and gefitinib-WBRT groups were longer than in the WBRT group, suggesting the superiority of combination therapy over standard WBRT. Moreover, bevacizumab-gefitinib-WBRT appeared to be more favorable than gefitinib-WBRT since it had a longer median OS time. A retrospective study showed that gefitinib-WBRT was appropriate for BM patients who were treated with EGFR-tyrosine kinase inhibitors (TKIs) (30). Another phase II clinical study indicated that the median OS time in patients who suffered from BM resulting from NSCLC was substantially improved by gefitinib-WBRT, which was consistent with our conclusions (31).

It has been documented that genetic mutations of EGFR appeared closely correlated with sensitivity of EGFR-TKI. Compared with NSCLC patients carrying normal EGFR genotypes, subjects carrying EGFR mutations (64.7%) were remarkably more sensitive with longer time to progression (21.7 *vs* 1.8 months) and OS (30.5 *vs* 6.6 months) after treatment with gefitinib. Nevertheless, certain NSCLC patients without EGFR mutations could still achieve PR, suggesting that EGFR mutations might not explain all cases of gefitinib efficacy (32).

As suggested by higher RR and DCR, both bevacizumab-gefitinib-WBRT and gefitinib-WBRT were more effective than standard WBRT, and bevacizumab-gefitinib-WBRT was ranked the most effective treatment since it had the highest RR and DCR. As suggested by a prospective phase II study on gefitinib-WBRT treatment, the RR and DCR of patients with BM resulting from NSCLC were approximately 81% and 95%, respectively, whereas the median PFS and OS time were 10 months and 13 months, respectively (33). An important finding from Park et al. suggested that gefitinib might enhance cell sensitivity, which has a significant impact on radiation effectiveness for treating A549 cell lines in lung cancer (34). Furthermore, another study revealed that gefitinib-WBRT treatment inhibited synergistic tumor growth in SCC-1 xenograft models (35), and WBRT may effectively increase the concentration of gefitinib in the central nervous system (30). When brain or meningeal metastasis occur, incompleteness of tumor angiogenesis and tumor edema would contribute to destruction of the blood-brain barrier, making it easier for TKIs to pass through the barrier raising the concentration of TKIs within cerebral spinal fluid. Although the concentration of TKIs within cerebral spinal fluid was shown to be below that within serum, its anti-tumor activity appeared to overweigh the diffusivity within metastasis foci targeted by treatment of BM (32,36).

As a molecular-targeted drug, bevacizumab possessed serious toxic side effects, which could be eliminated by reducing the dosage or discontinuing the drug. The most common adverse effects of bevacizumab included fatigue (45%), hypertension (12–34%), proteinuria (4–36%), nasal bleeding (19–35%) and venous thromboembolism (8–21%) (37,38). Other relatively infrequent complications are cerebral hemorrhage, nephrotic syndrome, gastrointestinal perforation, cerebral ischemia, acute myocardial infarction, among others (39,40).

This clinical trial compared the effectiveness of bevacizumab-gefitinib-WBRT, gefitinib-WBRT and standard WBRT in order to verify the hypothesis that combined WBRT may contribute to more desirable survival status for patients with BM resulting from NSCLC. Several limitations were present in this study due to resource constraints. For instance, we did not estimate the optimal sample size for each treatment group, which may affect the statistical power of our study. Moreover, some clinical information of patients was missing, which was imputed based on clinical knowledge, and this may give biased results since we do not know whether this missing information was random or not. Third, it is still unknown how bevacizumab and gefitinib interact with WBRT to improve the overall efficacy of standard WBRT. As a result, we strongly encourage researchers to further explore the effectiveness and safety of bevacizumab-gefitinib-WBRT and gefitinib-WBRT.

In conclusion, introducing bevacizumab or gefitinib into standard WBRT significantly improved survival status of patients with BM resulting from NSCLC when compared with WBRT alone. Bevacizumab-gefitinib-WBRT resulted in better treatment efficacy than gefitinib-WBRT, providing solid evidence that the synergic combination of bevacizumab, gefitinib and WBRT may be clinically valuable for these patients.

## Supplementary material

Click here to view [pdf].
